# How can we discover the most valuable types of big data and artificial intelligence-based solutions? A methodology for the efficient development of the underlying analytics that improve care

**DOI:** 10.1186/s12911-021-01682-9

**Published:** 2021-11-29

**Authors:** Lytske Bakker, Jos Aarts, Carin Uyl-de Groot, Ken Redekop

**Affiliations:** 1 Erasmus School of Health Policy and Management, Erasmus University, P.O. Box 1738, 3000 DR Rotterdam, The Netherlands; 2 Institute for Medical Technology Assessment, Erasmus University, Rotterdam, The Netherlands; 3 Erasmus Centre for Health Economics Rotterdam (EsCHER), Erasmus University, Rotterdam, The Netherlands

**Keywords:** Analytics, Artificial intelligence, Big data, Cost–benefit analysis, Critical care, Chronic lymphocytic leukaemia

## Abstract

**Background:**

Much has been invested in big data and artificial intelligence-based solutions for healthcare. However, few applications have been implemented in clinical practice. Early economic evaluations can help to improve decision-making by developers of analytics underlying these solutions aiming to increase the likelihood of successful implementation, but recommendations about their use are lacking. The aim of this study was to develop and apply a framework that positions best practice methods for economic evaluations alongside development of analytics, thereby enabling developers to identify barriers to success and to select analytics worth further investments.

**Methods:**

The framework was developed using literature, recommendations for economic evaluations and by applying the framework to use cases (chronic lymphocytic leukaemia (CLL), intensive care, diabetes). First, the feasibility of developing clinically relevant analytics was assessed and critical barriers to successful development and implementation identified. Economic evaluations were then used to determine critical thresholds and guide investment decisions.

**Results:**

When using the framework to assist decision-making of developers of analytics, continuing development was not always feasible or worthwhile. Developing analytics for progressive CLL and diabetes was clinically relevant but not feasible with the data available. Alternatively, developing analytics for newly diagnosed CLL patients was feasible but continuing development was not considered worthwhile because the high drug costs made it economically unattractive for potential users. Alternatively, in the intensive care unit, analytics reduced mortality and per-patient costs when used to identify infections (− 0.5%, − €886) and to improve patient-ventilator interaction (− 3%, − €264). Both analytics have the potential to save money but the potential benefits of analytics that identify infections strongly depend on infection rate; a higher rate implies greater cost-savings.

**Conclusions:**

We present a framework that stimulates efficiency of development of analytics for big data and artificial intelligence-based solutions by selecting those applications of analytics for which development is feasible and worthwhile. For these applications, results from early economic evaluations can be used to guide investment decisions and identify critical requirements.

**Supplementary Information:**

The online version contains supplementary material available at 10.1186/s12911-021-01682-9.

## Background

With the increasing ability to collect healthcare data, billions of dollars have been invested in (big) data analytics and artificial intelligence (AI) by private (e.g. IBM, Google, hospitals) and public institutions worldwide (e.g. Agency for Healthcare Research and Quality, the Patient-Centered Outcomes Research Institute, European Commission) [1–9]. Analytics can be applied in many ways, and it has often been suggested that they can improve care for a wide variety of clinical fields [10–15]. Bates et al. define big data analytics as the discovery and communication of patterns in datasets that are extremely complex due to their size (volume), rapid collection (velocity) and/or the need to combine multiple data sources (variety) [14]. The term Artificial Intelligence was first mentioned many years ago and is defined as the ability of computers to mimic or simulate the human mind [16]. However, despite many publications on the potential of big data analytics and AI, few analytics have been implemented [6, 17–20] and resulted in health benefits and/or cost savings [21–23].

Data availability can be an important barrier to the development of analytics that improve healthcare [4, 12, 17, 24–26]. The datasets required to develop machine learning models should be large and, depending on the method used, should contain sufficient data on relevant features [11, 27]. Data-related problems mentioned in the literature include limited sample size [4, 24–26, 28], a short duration of follow-up [24], validity of results with heterogeneous patient populations and selection bias [4, 13, 17, 24, 28, 29] and bias due to missing data [12, 24, 29, 30]. Moreover, successful development does not mean easy implementation; important barriers to implementation include the need for prospective validation [4, 24, 28] and the high costs of validation and implementation [4, 19, 24, 31–33].

For other healthcare technologies, such as drugs, medical devices and diagnostic tests, economic evaluations are used to assess the potential impact of anticipated barriers early on during development [34–38]. In economic evaluations, the health benefits and costs of novel technologies are compared to the benefits and costs of an alternative such as current care. Use of these economic evaluations alongside development is recommended to assist decision-making by developers, to analyse the impact of uncertainty in performance of the technology on outcomes, and to identify critical requirements (e.g. price) for successful market access and dissemination [36, 37]. A key aim of this approach is to increase the likelihood of successful market uptake and avoid wasting investments due to failed implementation.

Very few economic evaluations of analytics exist [13, 17, 20–23, 39, 40] and the ones that do have omitted relevant costs [19, 22]. Moreover, recommendations on how and when to perform economic evaluations of analytics do not exist, even though their use could improve development efficiency by identifying analytics with the greatest potential health impact. In this paper, we present a framework that can assist developer decision-making by selecting applications of analytics that are not only worth developing but also feasible.

## Methods

**Fig. 1:**
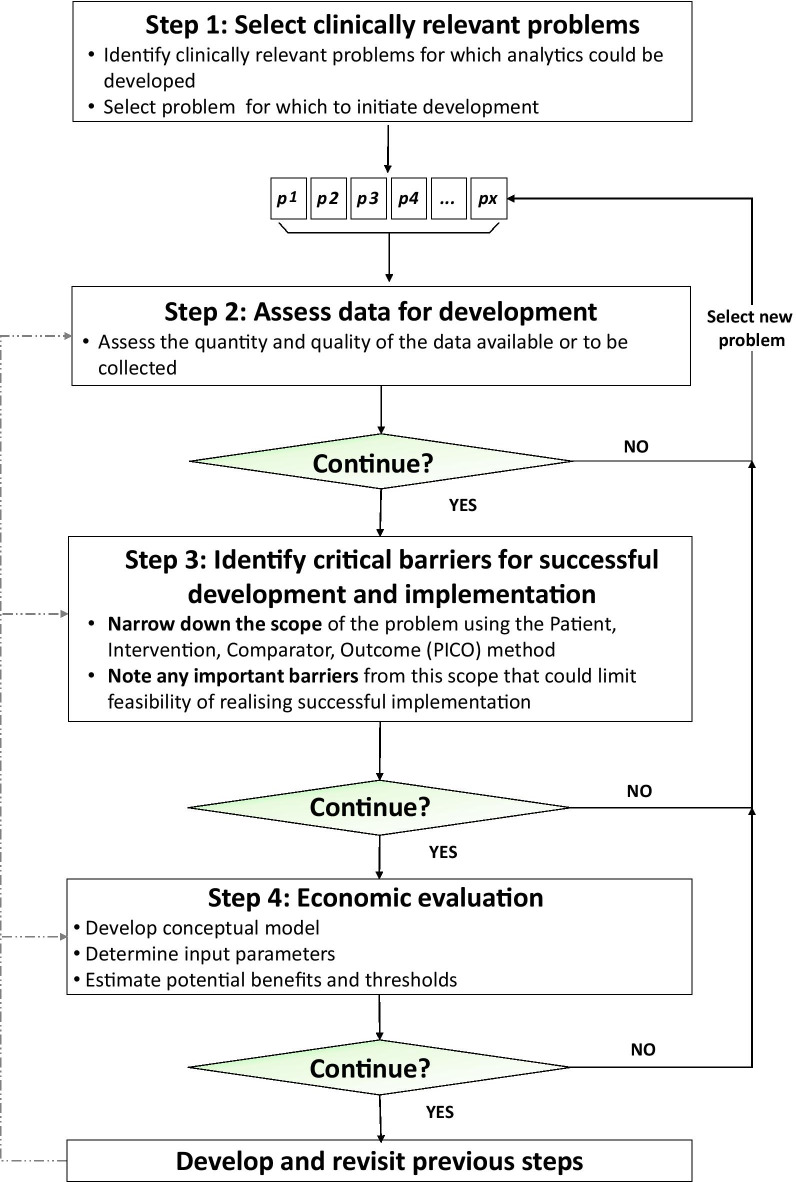
Flowchart for assessing health economic benefits of novel analytics alongside development. *p* = *problem*

We present a framework that efficiently selects analytics that are relevant, feasible and capable of generating important health and economic benefits (Fig. 1). The framework was developed based on challenges of analytics development defined in the literature and best practice recommendations for economic evaluations. It was then further refined by applying it in three clinical use cases. The use cases were selected from a European Horizon 2020 funded project (AEGLE) that aimed to develop a cloud-based big data analytics platform. The three use cases focused on chronic lymphocytic leukaemia (CLL), the intensive care unit (ICU) and diabetes.

### Step 1: select clinically relevant problems

This first step involves selecting relevant clinical problems. Whether problems are considered clinically relevant depends on the setting for which analytics are developed and the experts involved. When analytics are developed for a local hospital (i.e. for a learning health system), local experts should be consulted to identify relevant problems. When the aim is to develop analytics for a wider audience such as clinical experts in different countries or continents, then interviews with multiple potential users are recommended alongside a review of guidelines and the literature. Needless to say, a multidisciplinary approach throughout this step is crucial [10, 41].

### Step 2: assess data for development

After relevant problems are selected, it is necessary to assess whether the data available, or to be collected, is of sufficient quantity and quality to address the problem. Such an assessment may include careful scrutiny of the sample size, duration of follow-up, expected frequency of missing data, potential sources of bias and heterogeneity in care practices between sites. Moreover, the timing of data collection and the types of outcomes collected during follow-up may differ between clinical sites.

### Step 3: identify critical barriers to realising successful development and implementation

The scope of the problem should be narrowed down and used to identify critical barriers prior to estimating costs and benefits. Narrowing down the scope is a critical step in any economic evaluation [37] and one way to achieve this is through the Population (or Patient), Intervention, Comparator, and Outcomes (PICO) method [37]. First, the target population (P) is defined, which can include a description of the setting and the population size. The intervention (I) should include a description of the care pathways involved, including the analytics to be developed, the additional software and hardware needed to use the analytics, and the actions that follow from use of the analytics. The description of the comparator (C) entails a discussion on treatments available and relevant software and hardware elements used in current care. The final component of outcomes(O), refers not just to clinical outcomes but all outcomes considered relevant by users and purchasers, including mortality, life years gained, quality-adjusted life years gained(QALYs) and economic benefits. Ideally, they should go beyond diagnostic performance metrics like Area Under the Curve (AUC) [4, 17, 42, 43] and include outcomes related to health benefits, satisfaction and costs.

The detailed description of the scope, formulated using the PICO method, can then be used to identify potential barriers to successful development and implementation of the analytics. An example of a critical barrier is whether the health information system currently used in a health centre is sufficient to support the analytics or whether major upgrades are needed. If the examination of possible barriers does not reveal any insurmountable barriers, the health and economic benefits can be estimated. When continuing development seems risky, for instance because of the limited availability of required software and hardware elements in current practice, a developer can decide to select a new problem or cease development altogether.

### Step 4: economic evaluation

The next step is to perform an economic evaluation of the analytics that are considered feasible to develop. An evaluation starts by developing a conceptual model and collecting input data. A conceptual model can be developed in different ways, including the estimation of the number needed to treat [44], decision curve analysis [42, 43], decision trees, and Markov models. Depending on the stage of development, the models may vary from very simple to very complex. The validity of the model should be assessed according to best practice guidelines [37, 45]. Information on relevant input parameters required to populate the model can be collected alongside model development from sources such as patient-level data and the literature, but are sometimes limited to expert opinion or assumptions, particularly in the early stages of development. Uncertainty surrounding parameter estimates generally decreases as development progresses and more information becomes available [36, 38].

Base case estimates of potential benefits can then be determined using the most likely parameter values. Results can be presented using the incremental cost-effectiveness ratio (ICER) but more importantly; results should be presented such that they are understandable to the target audience (investors, future users and purchasers). The uncertainty in these point estimates should always be analysed using uncertainty analyses. Uncertainty analyses can include scenario analyses and sensitivity analyses, but also analyses to determine critical thresholds of relevant parameters, such as accuracy and pricing thresholds needed to realise health and economic benefits. The headroom can also be estimated according to the following formula:

$${\text{Headroom}} = {\text{N}} + \uplambda *{\text{Q}}$$

Here N refers to the potential savings where the costs of the technology are set to zero, λ is the willingness to pay threshold and Q are the health effects gained [46]. Moreover, probabilistic sensitivity analyses can be used to estimate the impact of uncertainty in all parameters simultaneously. For each parameter, random estimates are drawn many times (e.g., n = 1000) from their underlying distribution. For these estimates, the costs and effects are calculated and presented using a cost-effectiveness plane and a cost-effectiveness acceptability curve. In a cost-effectiveness acceptability curve, the probability that an intervention is cost-effective is plotted against a range of willingness to pay thresholds.

### Iterative approach

When a developer decides to continue development, the different steps (assess data for development, critical barriers to realising success, and the economic evaluation) should be revisited as needed throughout development, represented by the dotted line in Fig. 1.

### Clinical use cases

#### Chronic lymphocytic leukaemia

The first clinical use case, focused on developing cloud-based analytics using next generation sequencing (NGS) data of CLL patients from three clinical sites across Europe (Sweden, Italy & Greece). CLL is characterised by considerable heterogeneity in disease progression [47, 48] and after diagnosis, the majority of CLL patients are followed according to a ‘watch and wait’ (W&W) strategy. Roughly 60% of these patients progress to having active disease requiring treatment [47]. The treatment they receive depends on their molecular profile and general fitness as well as on treatment approval and availability [47].

#### Intensive care

In the second use case, the aim was to develop analytics for ICU care using routinely collected data. Data from electronic health records (EHRs) and mechanical ventilators of patients from a Greek ICU was available for development. There are many ways in which analytics can improve ICU care and a variety of applications have been suggested in the literature [10, 11]; these include analytics to determine readmission risk, predict length of stay, diagnose sepsis, and improve the interaction between patients and mechanical ventilators [11].

#### Diabetes mellitus (diabetes type 2)

Many diabetes treatments are available, and these can often be combined to improve effectiveness. However, evaluating efficacy for all combinations, types of patients and treatment lines in randomised controlled trials would not be feasible, and using EHRs to evaluate effectiveness of treatment combinations has previously been suggested [30]. In this third use case, the aim was to develop analytics using EHRs in the United Kingdom to personalise diabetes treatment for patients.

## Results

The framework was applied to three clinical use cases (e.g. CLL, intensive care and diabetes) (Table 1). The results for each case are described one by one.

**Table 1:** The methodology applied to address problems in care for chronic lymphocytic leukaemia, the intensive care and diabetes

	CLL Problem 1	CLL Problem 2	ICU Problem 1	ICU Problem 2	Diabetes
Clinically relevant problem	Variations in treatment response to 1st and 2nd line	Imperfect algorithms for identifying newly diagnosed, high-risk CLL patients	Identifying patients with ineffective efforts at risk of poor outcomes	Diagnosing catheter related bloodstream infections (CRBSI)	Unknown variation in response to treatment with SGLTs + GLPs
Assess data for development	– NGS data available – Follow-up probably sufficient – Large variation in treatments	– NGS data available – Follow-up sufficient	– Monitoring & EHR data available – Sufficient sample size, sufficient follow-up, limited missing data	– EHR & biosignal data available & continued prospectively – Limited missing data anticipated	– EHR data available from secondary care – Large amounts of missing follow-up data
Identify critical barriers for successful development and implementation	–	P: Newly diagnosed CLL patients without treatment indication I: Analytics that identify high risk patients followed by treatment with ibrutinib C: Stratification using clinical symptoms without receiving treatment O: Costs, LYG, QALYs Barriers: – Site-specific validation required – Reimbursement of novel treatment	P: Patients on assisted mechanical ventilation I: Identify patients at risk of poor outcomes with analytics and intervene to avoid ineffective efforts C: Care in which ineffective efforts are not identified O: Mortality, LOS, costs, LYG, QALYs Barriers: – Availability of monitor that identifies ineffective efforts – Site-specific validation	P: Patients with central venous catheter I: Early identification of CRBSI, catheter removal & antibiotics C: Late identification of CRBSI, catheter removal & antibiotics O: Mortality, LOS, costs, LYG, QALYs Barriers: – Varying prevalence of CRBSI – Integration of analytics in an EHR – Site-specific validation	–
Economic Evaluation	–	Benefits: 0.13 QALYs, + €89,985	Benefits: − 3% mortality, 0.21 QALYs, − €264 [58]	Benefits: − 0.5% mortality, + 0.06 QALYs, − €886	–
Continue development	Not feasible. Sample size too small and large variations in prescribing practices	Not feasible. High costs of treatment offset benefits gained	Feasible. Invest in research into the effectiveness of intervention and the price of the analytics [58]	Feasible. If the target market extends beyond Greece the impact of the prevalence of CRBSI on benefits should be considered	Not feasible. Small sample size and large amount of missing follow-up data

*CLL* chronic lymphocytic leukaemia, *ICU* intensive care unit, *NGS* next generation sequencing, *SGLTs* sodium glucose transporter-2 inhibitors, *GLPs* glucagon-like peptide-1 agonists, *CRBSI* catheter related bloodstream infection, *EHR* electronic health record, *LOS* length of stay, *LYG* life years gained, *QALY* quality-adjusted life years gained

### Case 1: CLL

Because of the heterogeneous nature of CLL progression and treatment response, stratifying patients according to their expected prognosis could improve care [47]. In discussions with clinical experts, problems were selected based on the three decision points suggested by Baliakas et al. The first is upon diagnosis, when clinicians want to determine which patients are likely to progress to active disease. The second decision point is the moment when patients have active disease, and a first-line treatment needs to be selected. The third is the decision point when first-line treatment has failed, and a decision needs to be made about which second-line treatment is best for a patient [47]. CLL experts stated that decision points two and three were the most clinically relevant.

Regarding decision point one, developing analytics to improve stratification for these patients was considered feasible with the data available (Table 1). In contrast, the feasibility regarding decision point two was limited because of large variations between countries in the treatments prescribed. For decision point three, development of analytics to improve decision-making would not be feasible because it was expected that few patients in the data set received second-line treatment, which therefore meant a small sample size. Consequently, the first decision point was considered the best choice for analytics development.

When defining critical barriers, the scope included newly diagnosed Swedish CLL patients. In current care, these patients are not treated, but are regularly seen by the haematologist and undergo a blood test. When developing the analytics in 2015–2016, no treatment was available for patients with a high risk of progression. The only possible changes in care available at the time was the ability to personalise the intensity of follow-up and the ability to inform patients about their risk. These very limited options of ‘treatment’ can be considered a critical barrier for success since it is likely that costs of NGS and analytics are high while health benefits could only be expected through the reduction in a patient’s uncertainty (and anxiety) regarding prognosis. Therefore, at the time, analytics development did not continue beyond research purposes. However, a recent publication has suggested that early treatment of intermediate- and high-risk patients with ibrutinib could delay time to next treatment. Given these new findings, we updated results for this application, including the possibility of treatment with ibrutinib as part of the intervention.

**Fig. 2:**
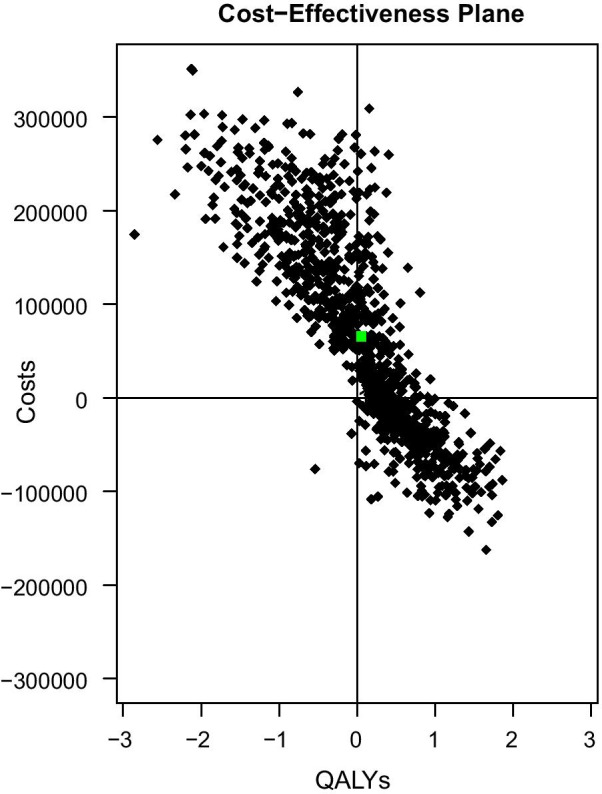
Cost-effectiveness plane reporting the quality-adjusted life years and costs (€) from the probabilistic sensitivity analysis

After the PICO question was formulated, input parameters (probabilities, utilities, unit costs and resource use) were derived from the literature, Swedish guidelines, and expert opinion (Additional file 1: Table S1). A four state Markov model (Additional file 1: Fig. S1) was used to estimate costs, life years and quality-adjusted life years adopting a lifetime time horizon and a healthcare payer perspective. Long-term survival was estimated by combining results on time to next treatment from Condoluci et al. [49] with the hazard ratio reported in preliminary results from a randomised controlled trial comparing early ibrutinib treatment with current care [50]. More details on the model structure and input parameters used to estimate the health and economic benefits can be found in the Additional file. Even if an effective treatment is available, it is unlikely that analytics to improve stratification of newly diagnosed watch and wait CLL patients would be considered cost-effective: use of analytics would lead to a substantial cost increase (€89,985) but only a modest gain in health (0.13 QALYs) (Table 2). We demonstrated the relevance of univariate uncertainty analyses to assess the impact of parameter uncertainty (Additional file 1: Fig. S2). In univariate uncertainty analyses, the impact of an individual parameter is assessed by varying its estimate while keeping all other parameters constant. Here, the high costs of the treatment in the intervention arm are decisive in the incremental costs. The relevance of scenario analyses is demonstrated in Table 2 where even in the best-case scenario, analytics are unlikely to be cost-effective, since the incremental cost-effectiveness ratio exceeds thresholds used in Sweden. When varying all parameters simultaneously in the probabilistic sensitivity analyses, most of the estimates are in the upper right and left quadrant (Fig. 2). This means that most estimates reflect higher costs and either higher or lower QALYs. When these results are shown on a cost-effectiveness acceptability curve, we can see that better stratification of watch and wait patients and subsequent treatment with ibrutinib has an extremely low chance of being cost-effective (Additional file 1: Fig. S3).

**Table 2:** Results from the base case and best case scenario for analytics to improve stratification of watch and wait patients in chronic lymphocytic leukaemia compared to current care

	Costs	Life years	QALYs
*Base case*			
Current care	€103,947	11.18	8.57
Care with analytics	€193,932	11.51	8.69
Incremental	€89,985	0.34	0.13
ICER	–	€268,373	€708,192
*Best case scenario*^a^			
Current care	€98,458	11.18	8.57
Care with analytics	€155,667	11.58	8.91
Incremental	€57,209	0.41	0.34
ICER	–	€141,972	€166,879

*ICER* incremental cost-effectiveness ratio

^a^Best case scenario = low HR of time to next treatment with early ibrutinib treatment (0.11), 50% reduction in costs of ibrutinib per cycle (€2542), 50% reduction of costs of venetoclax with 50% (€2731), low costs of analytics and genomic and genetic testing (€100), High quality of life for those receiving early treatment with ibrutinib (0.78)

### Case 2: the intensive care unit

For the intensive care, relevant problems were identified through discussions with an intensivist at the Greek hospital that was involved in development.

#### Catheter related bloodstream infection

The first ICU-related problem selected, was that infections caused by central venous catheters were often diagnosed only after they are severe. Catheter related bloodstream infections (CRBSIs) are considered an important issue in the ICU since infected patients have an increased mortality and prolonged length of stay compared to other ICU patients [51]. The aim was to use analytics to diagnose CRBSI in an early stage to reduce disease severity, risk of death and costs.

EHR and biosignal data were available to develop the analytics (N = 2000) and additional records were to be collected prospectively. The required follow-up was short, and the relevant parameters needed to develop the analytics and evaluate outcomes (e.g. mortality, length of stay) were routinely collected. Missing data was expected to be present but manageable.

No insurmountable barriers were identified when narrowing down the scope in the early stages of development. An example of a potential barrier for the CRBSI analytics is the uncertainty in the probability of CRBSI. The frequency of CRBSI varies tremendously across countries and sites. In Western European countries, the reported incidence of CRBSI is low [52]. However, for the Greek hospital for which analytics were developed 7.5% of patients developed CRBSI during their ICU stay [53] and in other Greek hospitals reported even higher percentages (22.4%) [54]. If the target market for the analytics would have been limited to the US and western European countries, obtaining better estimates of the frequency of CRBSI would have been recommended prior to continuing with an economic evaluation. Another barrier might have been the need for EHRs to enable the analytics. However, since most Greek and European hospitals have adopted EHRs this was not expected to be an issue. Additional validation when adopting results in other hospitals would probably be required and feasible but would need to be taken into account in the economic evaluation. Based on these barriers, continuing with the economic evaluation was recommended.

**Fig. 3:**
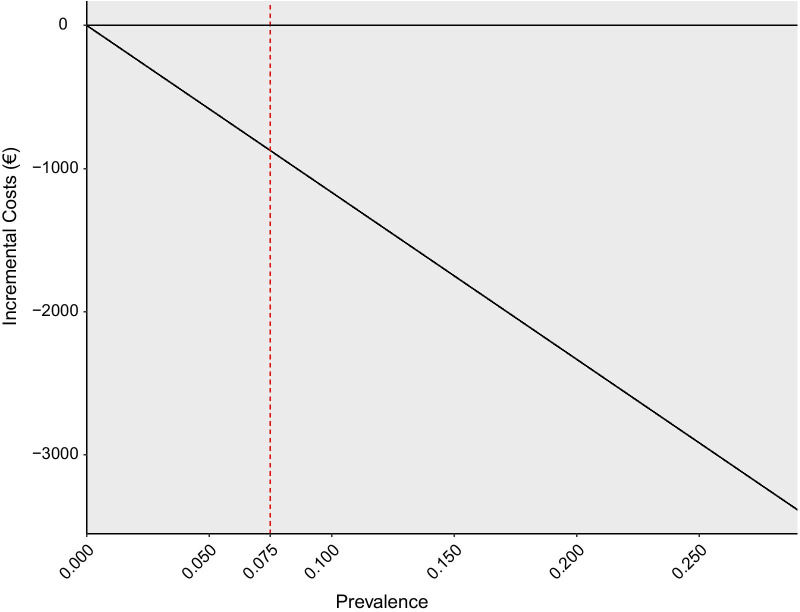
Impact of the prevalence of catheter related bloodstream infection in the intensive care unit on incremental savings

A detailed description of the model and input parameters used to estimate health and economic benefits can be found in Additional file 1: Fig. S4 and Additional file 1: Table S2. A decision tree was combined with a four state Markov model (Additional file 1: Fig. S4), adopting a lifetime time horizon and including only direct medical costs. Input parameters were derived from the literature, hospital reports, and expert opinion. The effect of earlier intervention on ICU mortality and ICU length of stay were derived from a study reporting the effect of earlier prescription of antibiotics [55]. Initial estimates demonstrated that continuing development was worthwhile since analytics could reduce mortality (0.5%), improve QALYs (0.06) and lead to cost-savings (€886) per patient. All input parameters were varied extensively in uncertainty analyses but the probability of CRBSI had substantial influence on the results. When the price of the technology was below €19,216 per bed, the analytics could reduce costs compared to current care. This meant that the headroom to achieve cost-neutrality with the intervention was €19,216 per bed, which meant there was sufficient room for costs of analytics, validation, and implementation. Given the large potential for the analytics to generate savings it was considered relevant to continue with development. However, the key factor that influenced benefits was the prevalence of CRBSI (Fig. 3). In this case, it was worthwhile to closely monitor site-specific prevalence throughout development and carefully consider the appropriate target market given the large variation in prevalence across sites.

#### Ineffective effort events

The second ICU-related problem to be addressed with analytics, was suboptimal interaction between patients and their mechanical ventilator. One form of suboptimal interaction relates to ineffective efforts where a patient tries, but fails, to trigger the mechanical ventilator into providing a breath. Several studies have found that ineffective efforts could be associated with worse outcomes [56, 57]. Here the aim was to enable clinicians to intervene in those patients with ineffective efforts, who are therefore at risk of having worse outcomes.

EHR records were available for all patients and once again relevant parameters were routinely collected and missing data was expected to be manageable. Furthermore, recordings of > 24 h for more than 100 patients were available from a prototype monitor detecting patient-ventilator interaction.

When assessing feasibility, no barriers were considered insurmountable (Table 1). An important barrier was the need to have a monitor capable of measuring ineffective efforts in addition to analytics that could identify patients with ineffective efforts at risk of having worse outcomes. The prototype monitor available in the Greek ICU would need to be purchased in order to use the analytics. Furthermore, costs of site-specific validation would need to be included in the economic evaluation.

The model and input parameters used to estimate the health and economic benefits have been previously reported [58]. The potential impact of analytics that identify patients with ineffective efforts at risk of having worse outcomes also suggests that continuing further development is worthwhile [58] since it can reduce mortality by 3%, increase QALYs by 0.21 and reduce costs (€264) [58]. Furthermore, it was demonstrated that even if the effectiveness of intervening was varied extensively, benefits could still be achieved [58]. The headroom for the analytics to generate savings (€7307) was considered sufficient to cover relevant hardware costs and additional costs of site-specific validation. Thus, further development was considered both relevant and feasible and the potential impact of the analytics was considered substantial.

### Case 3: diabetes mellitus

For diabetes, clinicians indicated that a highly relevant problem was to determine predictors of response to treatment with sodium glucose transporter-2 inhibitors combined with glucagon-like peptide-1 agonists. EHR data was available from diabetes patients treated in secondary care in the United Kingdom. However, a small sample size and substantial missing follow-up data raised questions about the feasibility of development, which resulted in the decision not to assess critical barriers and conduct an economic evaluation.

## Discussion

In this paper, we present a framework that aims to promote the efficient development of high potential analytics by rapidly assessing whether it is feasible and worthwhile to continue development. The use cases demonstrate the value of first assessing the feasibility of development and identifying relevant barriers before estimating the potential health and economic benefits of analytics. Examples were presented for CLL and diabetes where development was not feasible given the data available. Furthermore, the essence of critically narrowing down the scope is demonstrated for CLL and the ICU where the absence of actionable output is an important barrier to realising success and disease prevalence strongly influences benefits.

Early economic evaluations of analytics can assist decision-making of developers and stimulates them to develop those analytics with the greatest potential benefits. These evaluations allow developers to assess the influence of certain requirements of analytics (e.g. the costs of the technology, validation and implementation) on their potential health and economic impact. In our use cases, we see risks that could strongly influence widespread adoption, such as the prevalence of CRBSI and the high drug costs for CLL. Early economic evaluations can also be used to strengthen the business case of developers seeking funding for prospective validation and evaluation. This is especially relevant since the high costs of validation and implementation are important barriers to successful use of analytics in clinical practice [4, 19, 24, 31–33]. During implementation, data and tools used to perform early economic evaluations alongside development can be reused to perform a ‘late’ economic evaluation to convince payers that the analytics are worth purchasing. Elements covered in this framework align with key economic information sought by payers such as the UK’s National Institute for Health and Clinical Excellence [59].

However, for efficient development, economic evaluations should only be initiated for those applications deemed feasible and after ensuring that there are no critical barriers to success. Often multiple analytics can be developed for a single setting, disease or using a single dataset [27, 60]. For instance, for the ICU [11] and diabetes care [61] many more types of EHR-based analytics have been suggested than the ones presented here. This is an important difference compared to when early economic evaluations are used to assist decision-making during development of a technology with one or few applications (e.g. diagnostics). Since it is often unrealistic to evaluate—all potential applications of a particular type of analytics, our framework stimulates developers to select which applications are worthy of additional resources. Where feasibility is clearly a problem for the diabetes use case, the lack of an actionable output is the shortcoming for CLL; an issue often reported in the literature [10, 15, 24, 25]. The initial analyses performed in the early economic evaluation can be very simple at first but can become more complex as development progresses; this corresponds with recommendations that analytics development and validation should also be iterative [4, 62]. However, as with analytics for CLL and CRBSI, it is sometimes worthwhile to invest more time in adding additional details at an early stage, since it is better to fail fast when limited investments have been made. Using early economic evaluations in an iterative manner and providing a detailed definition of the scope aligns with best practices for early economic evaluations of other healthcare technologies such as diagnostic tests [34–38]. The recommendations provided by others such as Drummond et al. [63], or Buisman et al. [37] regarding the selection of a model structure (e.g. decision tree, Markov model), estimation of input parameters, and calculating outcomes (such as the ICER) are likely to be applicable when estimating benefits. We demonstrate in the CLL and diabetes use cases how the framework may assist developers in selecting those applications that are likely to succeed, before investing additional resources in performing an economic evaluation. Similar to other papers [e.g. 4, 12, 17, 24–26], we found the data available for development to be a barrier to success in the CLL and diabetes case studies. Analytics for artificial intelligence are ‘data hungry’ and therefore require large datasets [11, 27]. Furthermore, the quality of the data is an important issue when developing and using AI. Roberts et al. have emphasised in their review of AI for the diagnosis and prognostication of secondary pneumonia, that many AI analytics were hampered by poor quality data [64]. Our framework aligns with recommendations by Vollmer et al. who include critical questions regarding the data used as part of their framework to inform design and evaluate AI analytics [65]. Reviewing the data quality ensures developers select those applications of analytics for which development is most likely to succeed. For instance, rapid checks of potential sample sizes have been previously suggested [66]. For analytics with adequate data quality, additional resources can then be invested to perform an economic evaluation.

In this study, the framework was applied to three clinical use cases. Therefore, validation in other use cases is recommended. Other use cases can include different clinical areas (e.g. psychiatric disorders) but also other data sources such as data from patient devices (e.g. Fitbits), imaging and social media. Additional research could also assess criteria to value the quality of unstructured data. Furthermore, the framework presented could be easily adopted alongside initiatives such as RE-AIM used to translate research into practice [67]. This framework pays particular attention to the timing of economic evaluations intended to assist development considering relevant elements in the ‘Reach’, ‘Effectiveness’, ‘Adoption’, ‘Implementation’ and ‘Maintenance’ steps.

Since many factors can influence the successful implementation and adoption of analytics, we may have adopted a somewhat narrow approach by solely focusing on the value of economic evaluations to support developer decision-making. A wider form of decision support can be achieved through a broader evaluation of analytics, for instance using health technology assessment, which includes social, and ethical elements besides the health and economic impact [68]. Moreover, elicitation of stakeholder preferences such as patients and clinicians could ensure that potential barriers to development, acceptability and implementation are addressed [69].

In recent years, there has been an increased interest in the ethical challenges that we face relating to the adoption of artificial intelligence [70]. In this paper, we discuss that factors such as the risk of bias and small sample sizes, should be assessed at an early stage of development prior to performing an economic evaluation. Trocin et al. emphasise the severity of the consequences of failing to do so. Some of the challenges relating to the data quality mentioned in this paper have also been emphasised by Trocin et al. Moreover, these authors also provide research questions that need to be answered to ensure the responsible adoption of AI related technologies [70]. Many answers to these questions could be very relevant for future improvements of the flowchart. Depending on the setting and type of analytics, for instance, the quality of the data can be assessed according to the risk of selection bias in the data [4, 13], or the absence of ethnic variation in the data which could limit generalisability of machine learning models [4, 17, 28].

## Conclusions

This is the first study providing recommendations on the use of economic evaluations to support development decisions of analytics for big data and artificial intelligence-based solutions. Many types of analytics can be developed within a specific clinical setting or disease or using a particular dataset. The framework presented in this study stimulates efficiency of development by selecting those applications worth further investment after assessing the feasibility of development and identifying critical barriers. For these applications, early economic evaluations can assist decision-making of analytics developers by estimating for instance requirements of effectiveness and the headroom for pricing, validation, and implementation.

## Supplementary Information

**Additional file 1:** Description of model structure, input parameters and results for the example use cases.

## Data Availability

Not applicable.

## Abbreviations

AI: Artificial intelligence
AUC: Area under the curve
CLL: Chronic lymphocytic leukaemia
CRBSI: Catheter related bloodstream infection
EHR: Electronic health record
ICU: Intensive care unit
NGS: Next generation sequencing
PICO: Population, intervention, comparator, and outcomes
QALYs: Quality-adjusted life years
W&W: Watch and wait
