# Supplementary material for: How can we discover the most valuable types of big data and artificial intelligence-based solutions? A methodology for the efficient development of the underlying analytics that improve care
Source: BMC Med Inform Decis Mak. 2021 Nov 29;21:336. doi: 10.1186/s12911-021-01682-9 (PMC8628451; doi:10.1186/s12911-021-01682-9)
Supplement: Supplementary file 1 — Additional file 1: Description of model structure, input parameters and results for the example use cases. [file 12911_2021_1682_MOESM1_ESM.docx]

Appendix: How can we discover the most valuable types of big data and artificial intelligence-based solutions? A methodology for the efficient development of the underlying analytics that improve care.

Lytske Bakker, Jos Aarts, Carin Uyl-de Groot, Ken Redekop

**Content**

Model & Input parameters case study 1……………………….……………………………………………………….2-5

Figure S2: Tornado diagram for case study 1……………….…………………………………………………………..6

Figure S3: Cost-effectiveness acceptability curve for case study 1……..…………………………………….7

Model & Input parameters case study 2…..……………….………………………………………………………..8-13

**Model & Input parameters case study 1: Chronic lymphocytic leukaemia**

Several risk scores for newly diagnosed chronic lymphocytic leukaemia (CLL) patients are available that combine clinical, laboratory and/or molecular data to stratify patients according to risk of progression and time to treatment [1,2,3]. After diagnosis, patients without clinically active disease are monitored during frequent follow-up visits also referred to as the ‘watch & wait’ phase. During the watch and wait phase, prognostic scores are currently used to identify patients with a higher risk of progressing eligible for enrollment in clinical trials and to personalise their frequency of follow-up. Even though at present, no early treatment is prescribed to patients at higher risk of progressing, preliminary results from the CLL-12 study suggest that some patients without active disease might benefit from early treatment with ibrutinib [4].

In light of these recent results, we estimated the potential cost-effectiveness of using next generation sequencing data to improve prognostic algorithms for assessing the risk of progressing to needing treatment of patients diagnosed with CLL. Even though development to improve available risk scores is recommended [1], the potential health and economic benefits of using them have not yet been assessed. Prior to continuing development of analytics, it can be estimated whether further research into this area could be considered a worthwhile investment given this novel treatment available. The patient population consisted of patients newly diagnosed with CLL in a Swedish healthcare setting. Currently, these patients can be classified as high, intermediate or low-risk by assessing their unmutated immunoglobulin heavy variable gene status (IGHV), the absolute lymphocyte count, and the presence of palpable lymph nodes [1]. These patients are followed through a watch & wait strategy in which they receive frequent follow-up visits but do not receive treatment until the disease becomes clinically active. At present, no additional genomic or genetic testing is performed. In care with the novel analytics, those with a high and intermediate risk score would receive early treatment with ibrutinib.

**Figure S1:** Markov model used to estimate costs and effects of using analytics to estimate the risk of progression of watch and wait patients with chronic lymphocytic leukaemia compared to current care. W&W= watch and wait patients

We used a Markov model with 4 states (Figure S1) to estimate costs, life years gained and quality adjusted life years gained (QALYs). Tunnel states in the first-line health state were used to vary costs according to the respective time on treatment for the different treatments prescribed. A lifetime time horizon was adopted and the cycle length was 28 days. Even though a societal perspective has been recommended in Swedish guidelines for performing economic evaluations, this is not reflected in recent reimbursement decisions for CLL treatments. Here, cost-effectiveness was assessed by the TLV, The Dental and Pharmaceutical Benefits Agency in Sweden that decides on reimbursement decisions, without considering non-medical costs [5]. Therefore, we adopted a healthcare payer perspective including only direct medical costs. A discount rate of 3% was used for both costs and effects.

**Transition Probabilities**

Time to first treatment in current care was estimated using the survival curves presented in the supplemental figures of Condoluci et al [1]. Individual patient data was reconstructed according to Guyot et al. using Digizeit [6]. Care with the analytics assumed perfect stratification of patients where those progressing within 3 years were considered high risk, those progressing within 3-7 years are intermediate risk and those progressing after 7 years would be considered low risk. Background mortality in Sweden was used for the transition probabilities from watch and wait to death and first-line to death [7,8]. Sylvan et al. found that for 80% of patients in Sweden, treatments were prescribed in accordance with national guidelines [9]. Therefore, the first-line treatment in current care depended on the prevalence of IGHV mutations, Tp53 mutations and age and fitness of patients, in accordance with Swedish guidelines [10]. For the treatments prescribed in first- and second-line, the probability of requiring novel treatment over the first 24 months was derived from the time to next treatment curves and converted to rates to estimate probabilities in accordance with a 28-day cycle length. For second-line treatment in current care, overall survival curves were used to estimate the 28-day transition probability of death.

**Utilities**

Utility of watch and wait patients was derived from a study by Holtzer-Goor et al. [11] while utility values from Kosmas et al. [12] were used for utility of watch and wait patients receiving oral treatment and for patients with progressive disease.

**Costs**

When available, costs were based on estimates from studies and reports for the Swedish health care setting. The majority of unit costs for treatment in the first-line were obtained from a recent Swedish drug approval report for venetoclax from the Swedish HTA organisation [5]. Based on recommendations from the Svenska KLL Gruppen, it was assumed that in the progression state in current care 50% of patients received Ibrutinib, 20% received treatment with FCR and 30% received monotherapy with venetoclax [10]. Costs of progression in the intervention arm (€1,572) were based on rituximab treatment since patients have already received ibrutinib and venetoclax. Costs of analytics were obtained from a micro-costing study performed by Swarzche et al reporting the costs of genomic testing [13]. All costs were reported in 2019 euros.

**Cost-effectiveness analysis**

The base case input parameters were used to estimate the incremental cost effectiveness ratio. The incremental cost-effectiveness ratio is the incremental costs of the novel intervention compared to current care divided by the incremental effects. Hereafter, input parameters were varied extensively in univariate sensitivity analyses and scenario analyses. All parameters were varied simultaneously in the probabilistic sensitivity analysis using a beta distribution for probabilities and utilities and a gamma distribution for costs. R v3.6.3 was used for the model according to best practice modelling recommendations [14].

**Table S1**: Input parameters used to estimate costs and effects of using analytics to estimate the risk of progression of watch and wait patients with chronic lymphocytic leukaemia compared to current care. The values for all input parameters were obtained from the literature.

| Parameter | Base case | Distribution | Parameters | Lower | Upper | Source |
| --- | --- | --- | --- | --- | --- | --- |
| Probabilities |  |  |  |  |  |  |
| Patients classified in current care  Low risk  Intermediate risk  High risk | 0.24  0.38  0.38 | Dirichlet | Shape=0.24, 0.38, 0.38 |  |  | [1] |
| Patients classified with analytics  Low risk  Intermediate risk  High risk | 0.57  0.17  0.26 | Dirichlet | Shape= 0.57, 0.17, 0.26 |  |  | Perfect stratification assumed |
| HR Time to Next Treatment Ibrutinib in W&W vs placebo | 0.21 | Betapert | upper=0.39, lower=0.11, mode=0.21 | 0.11 | 0.39 | [4] |
| TP 1^st^-line FCR | 0.0050  Based on time to next treatment. Included as the first-line in current care for 24% of patients with mutated IGHV, fit, young patients and 8% of patients with unmutated IGHV, fit, young patients | Beta | se=0.0002 | 0.0044 | 0.0054 | [1,15,16] |
| TP 1^st^-line ChlO | 0.0130  Based on time to next treatment. Included as the first-line in current care for 44% of patients mutated IGHV, elderly, unfit patients | Beta | se=0.0007 | 0.012 | 0.014 | [1,16,17] |
| TP 1^st^-line VO | 0.0067  Based on time to next treatment. Included as the first-line in current care for 15% of patients in current care with unmutated IGHV, elderly, unfit patients | Beta | se=0.0003 | 0.0061 | 0.0074 | [1,16, 18] |
| TP 1^st^-line Ibrutinib | 0.0061  Based on time to next treatment. Included as the first-line in current care for 9% of patients current care with 17pdel / TP53 mutation | Beta | se=0.0003 | 0.0056 | 0.0068 | [10,19,20] |
| TP 1^st^-line VR | 0.0056  Based on time to next treatment. Assumed applicable to all patients. First-line for all patients in intervention arm | Beta | se=0.0003 | 0.0050 | 0.0061 | [21,22] |
| TP progression current care | 0.0107  Based on 50% of patients treated with ibrutinib, 30% of patients treated with venetoclax and 20% of patients treated with FCR | Beta | se=0.0005 | 0.0097 | 0.0118 | [10,23,24,25] |
| TP progression intervention | 0.075 | Beta | se=0.0038 | 0.068 | 0.083 | [26] |
|  |  |  |  |  |  |  |
| Utilities | | | | | | |
| Utility W&W | 0.81 | Beta | se=0.04 | 0.72 | 0.88 | [11] |
| Utility W&W on treatment | 0.71 | Beta | se=0.04 | 0.64 | 0.78 | [12] |
| Utility 1^st^ line | 0.71 | Beta | se=0.04 | 0.64 | 0.78 | [12] |
| Utility Progression | 0.66 | Beta | se=0.03 | 0.59 | 0.72 | [12] |
|  |  |  |  |  |  |  |
| Costs | | | | | | |
| Costs of next generation sequencing as diagnostic tool | €7439 per case  (€3719 per genome) | Gamma | Se=€1960 | €100 | €11,502 | [13] |
| Watch & wait health state cycle 1 to 6 | €45  Based on a consult with a hematologist and laboratory tests during the consult 6 months after diagnosis and a complete blood count every 2 months. | Gamma | Se=€11 | €26 | €69 | [27,28, expert opinion] |
| Watch & wait health state remaining cycles | €22  Based on a consult with a hematologist and laboratory tests during the consult every 15 months and a complete blood count every 2 months. | Gamma | Se=€5 | €13 | €34 | [27,28, expert opinion] |
| Fludarabine | €271 | Gamma | Se=€68 | €155 | €418 | [5] |
| Cyclophosphamide | €8 | Gamma | Se=€2 | €4 | €12 | [5] |
| Rituxumab | €773 | Gamma | Se=€193 | €442 | €1195 | [5] |
| Chlorambucil | €66 | Gamma | Se=€16 | €38 | €102 | [5] |
| Obinituzumab | €2393 | Gamma | Se=€598 | €1368 | €3700 | [5] |
| Ibrutinib | €3,813 | Gamma | Se=953 | €2180 | €5,897 | [5] |
| Venetoclax | €4096 | Gamma | Se=1024 | €2341 | €6334 | [5] |
| Adverse Events FCR | €643 | Gamma | se=€161 | €367 | €994 | [5] |
| Adverse Events ChlO | €304 | Gamma | Se=€76 | €174 | €470 | [5] |
| Adverse Events VO | €733 | Gamma | Se=€183 | €419 | €1133 | [5] |
| Adverse Events Ibrutinib | €804 | Gamma | Se=€201 | €459 | €1243 | [5] |
| Administration costs | €386 | Gamma | Se=€97 | €221 | €598 | [5] |
| TLS prevention | €2009 | Gamma | Se=€502 | €1149 | €3107 | [5] |
| Progression Current Care | €2,579  Based on 50% of patients treated with ibrutinib, 30% of patients treated with venetoclax and 20% of patients treated with FCR and follow-up costs. | Gamma | Se=€645 | €1,474 | €3,988 | [10,23,24,25,29] |
| Progression Intervention | € 1,572  Based on all patients receiving monotherapy with Rituxumab and follow-up costs. | Gamma | Se=393 | €899 | €2,431 | [29,30] |
| Follow-up first-line | €145 | Gamma | Se=€36 | €83 | €224 | [29] |
| Follow-up second-line | €780 | Gamma | Se=195 | €446 | €1207 | [29,30] |
| HR= Hazard Ration, TP= Transition Probability, W&W= Watch and Wait, FCR= Fludarabine + Cyclophosphamide+ Rituximab, ChlO= Chlorambucil + Obinutuzumab, VO= Venetoclax + Obinutuzumab, VR= Venetoclax + Rituximab, TLS= Tumor Lysis Syndrome | | | | | | |

**Figure S2**: Tornado diagram for incremental costs when using analytics to estimate the risk of progression of watch and wait patients with chronic lymphocytic leukaemia compared to current care. W&W= watch & wait patients, VR= venetoclax-rituximab


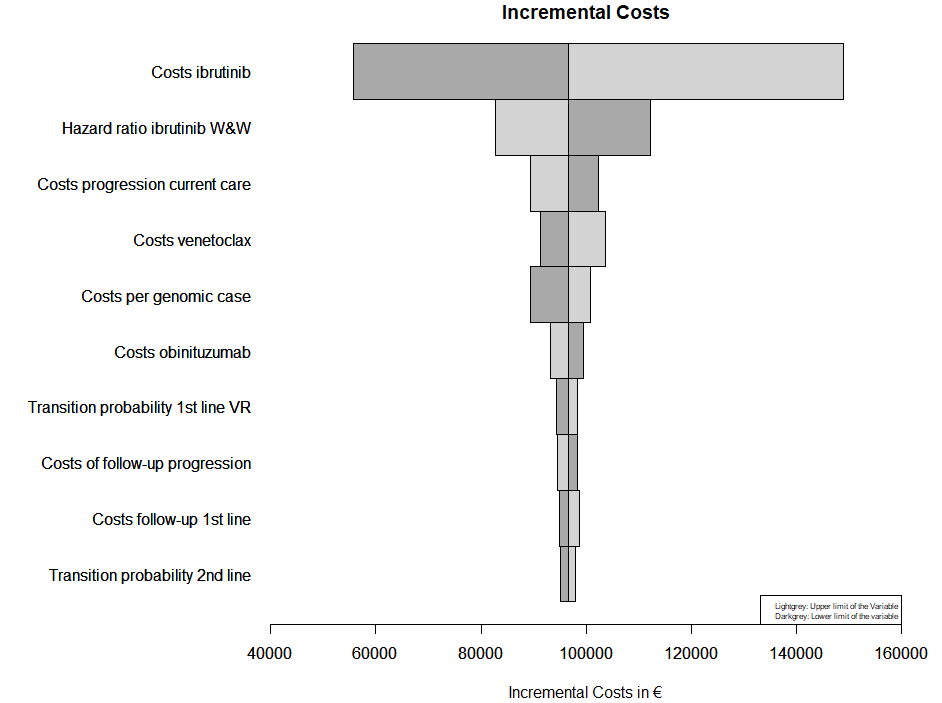


**Figure S3.** Cost-effectiveness acceptability curve with on the X-axis a range of willingness-to-pay thresholds and on the Y-axis the probability that the analytics and subsequent treatment with Ibrutinib would be cost-effective.


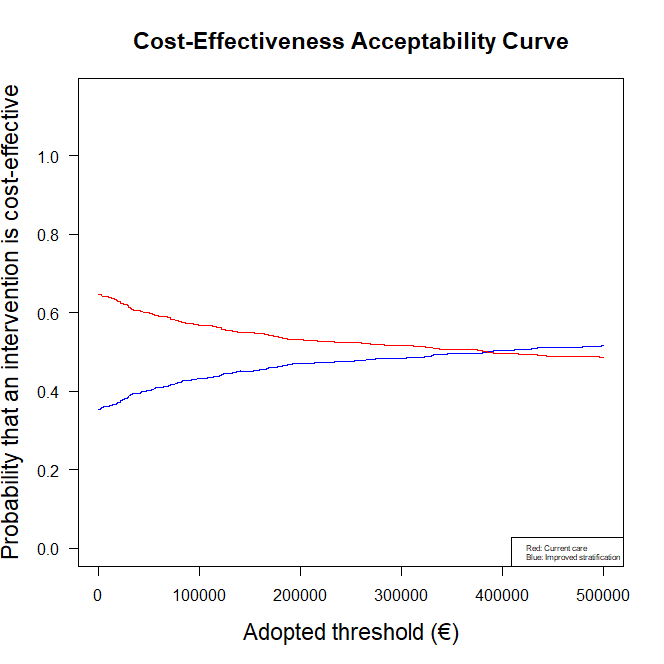


**Model & Input parameters case study 2: Catheter related bloodstream infection**

Infections are a recurring issue in the intensive care unit and can result in sepsis and septic shock. A common cause of these infections in Greece is placement of a central venous catheter [31]. Catheter related bloodstream infections (CRBSI) have been associated with increased mortality, prolonged intensive care unit (ICU) and hospital stay and prolonged mechanical ventilation [31,32]. Furthermore, CRBSIs are relatively easy to avoid by timely identification and infection control [33].

A decision tree model was combined with a four state Markov model to estimate the health and economic benefits of using analytics for earlier detection of CRBSI compared to current care in patients admitted to the ICU (Figure S4). In the decision tree, patients could receive care where CRBSI is diagnosed early with novel analytics (Arms 1- Arm7) or current care in which CRBSI was diagnosed according to clinical symptoms (Arm 8 -Arm 14). The diagnosis of CRBSI in current care is primarily based on clinical symptoms whereas when using analytics, CRBSI would be diagnosed in real-time. After diagnosis, the interventions for current care and care with analytics are identical; the catheter is replaced, and antibiotics are administered. In care with analytics, this intervention would be administered earlier due to a timely diagnosis. Patients falsely classified as having CRBSI would have their catheter replaced and receive antibiotics unnecessarily. False negatives would progress to having clear clinical symptoms resulting in a delayed diagnosis for which outcomes were assumed to be identical to patients in current care. When replacing the catheter, a subset of patients experienced complications such as pneumothorax, hematoma, and arterial puncture.

The decision tree ended in a Markov model in which all patients transitioned from ICU care to the general ward and were then discharged from the hospital. Cycle length for both of these states was identical to the median length of stay. For the post discharge state, a yearly cycle length was adopted. The possibility to return from discharge to the hospital ward state was not included given that there is no conclusive evidence that 30-day readmission rates are higher in patients with CRBSI [34]. Relevant decision-makers were hospital employees such as clinicians and budget managers in Greece. With these decision-makers in mind relevant health outcomes modelled were ICU length of stay, mortality, life years gained, and quality-adjusted life years gained (QALYs). Furthermore, a healthcare payer perspective was adopted including only direct medical costs. A discount rate of 3.5% was used for both costs and effects since national guidelines for performing economic evaluations in Greece are lacking. All analyses were performed using R v3.6.3.

**Probabilities**

All input parameters can be found in Table S2. The prevalence of CRBSI was based on an earlier report for the collaborating ICU in Greece (7.6%)[35]. Uncertainty surrounding these estimates was based on the large variation reported in the literature (0.5%-29%) [31,32]. In current care, the diagnosis is made according to clinical symptoms and the accuracy of the diagnosis in current care is uncertain. Sensitivity was high given that the diagnosis was made at a late stage at which clinical symptoms were clearly present whereas specificity was much lower. Sensitivity and specificity of the analytics were considered to be at least as good as algorithms already available in the literature (sensitivity= 85%, specificity= 83% [36,37]).

**Figure S4**: Decision tree and Markov model used to estimate costs and effects of using analytics for earlier identification of catheter related bloodstream infection compared to current care. CRI=Catheter related Infection, TP= True Positive, FP= False Positive, FN=False Negative, TN= True Negative, ICU= Intensive Care Unit.

The probabilities of ICU and hospital mortality were obtained from a large multicentre study following patients with CRBSI (28.5%) and without CRBSI (19.6%) [38]. We adopted a conservative approach in which we assumed that the analytics would result in earlier diagnosis and thus less severe outcomes instead of avoiding the CRBSI event altogether. We assumed that mortality with timely antibiotics resulted in a relative risk reduction in mortality (0.74) similar to the impact of early administration of antibiotics reported by Ferrer et al [39]. For survival after hospital discharge, the hazard ratio of dying after an ICU stay without CRBSI [40] and with CRBSI [41] were combined with survival in the Greek population [42,43]. The average incidence of complications across puncture sites was used from a Greek study [44] since all sites were used in the hospital in question.

**Utilities**

Utility estimates were obtained from the literature. No research is available reporting quality of life of patients during their ICU stay. Therefore, we adopted a utility estimate of 0.30 which corresponds to an EQ-5D state of extreme problems with selfcare, mobility and usual activities but no pain or discomfort assuming sedation was adequate. Quality of life after discharge was varied according to the time since hospital discharge and the mean age of the Greek patient population [45].

**Unit costs and resource use**

Length of stay in the ICU for patients with CRBSI (13) and without CRBSI (3) were obtained from Vught et al [38]. We assumed that ICU length of stay reduced with 24% with an early intervention, similar to the effect of early administration of antibiotics reported by Ferrer et al [39]. Length of stay after ICU discharge (15 days) was obtained from Vught et al. [38] and assumed to be identical for the intervention and current care. Patients with CRBSI in the model received treatment with antibiotics for 10.5 days [46]. This estimate was varied from 7-14 days. Costs of catheter replacement were based on a duration of change of 10 minutes. For the base case analysis, annual licensing costs per bed were included for the analytics (€959) [47]. Daily costs of antibiotics were derived from the literature [46] and unit costs of an ICU and hospital day were derived from Greek micro-costing studies [48,49]. Costs were reported in 2019 euros.

**Analyses**

For the base case estimate, incremental costs, length of stay, mortality, life years gained, QALYs and the incremental costs-effectiveness ratio were reported. Base case estimates were varied in univariate and probabilistic sensitivity analyses. In the probabilistic sensitivity analyses, all parameters were varied simultaneously except for the costs of the analytics. Underlying distributions adopted for probabilities were the beta and beta pert distribution. For costs and resource use, the gamma and beta pert distribution were used. We also estimated the headroom according to the following formula:

*Headroom = N + λ * Q*

Here N refers to the potential savings where the costs of the technology are set to zero, λ is the willingness-to-pay threshold and Q are the health effects gained [50]. Willingness-to-pay thresholds used were €4,946, €7,758 [51] and €30,000 [52,53]. We assumed patients occupied the bed for 7.4 days on average [35] and that the analytics should be functional for at least three years. At least 49 patients would be using the analytics each year. Costs of implementation were obtained from a systematic review that reported cost estimates for developing and implementing clinical decision support systems in EHRs for diabetes [54]. Costs of validation were based on recommendations reported by Calster et al. [55] for an ICU with 13 beds and a validation study including 100 patients.

**Table S2:** Input parameters used to estimate costs and effects of using analytics for earlier identification of catheter related bloodstream infection compared to current care. The values for the input parameters were obtained from the literature and if no evidence was available through discussions with experts and assumptions.

| **Parameter** | **Base Case estimate** | **Distribution** | **PSA** | **Lowest estimate** | **Highest estimate** | **Source** |
| --- | --- | --- | --- | --- | --- | --- |
| **Probabilities** | | | | | | |
| Prevalence of CRBSI | 7.6% | Beta pert | Min=0.5%, Max=29%, Mode=4.025% | 0.5% | 29% | [31,32,35] |
| Sensitivity CRBSI diagnosis Current Care | 100% |  |  |  |  | Assumption |
| Specificity CRBSI diagnosis Current Care | 60% | Beta pert | Min=40%, Max=100%, Mode=55% | 40% | 100% | Assumption |
| Sensitivity analytics | 85% | Beta pert | Min=75%, Max=100%, Mode=84% | 75% | 100% | [36,37] |
| Specificity analytics | 83% | Beta pert | Min=63%, Max=100%, Mode=84% | 63% | 100% | [36,37] |
| 30-day mortality without CRBSI | 19.6% | Beta | s.e.=1.96 | 15.9% | 23.6% | [38] |
| 30-day mortality with CRBSI | 28.5% | Beta | s.e.=2.85 | 23.1% | 34.2% | [38] |
| Relative risk of mortality with early intervention | 0.74 | Normal | s.e.=0.007 | 60% | 100% | [38,39] |
| Hazard ratio of mortality after discharge sepsis vs. no sepsis | 1.39 | Normal | s.e.=0.07 | 1.26 | 1.52 | [41] |
| Hazard ratio for survival after ICU discharge | 2.01 | Normal | s.e.=0.1 | 1.64 | 2.46 | [40] |
| Incidence of arterial puncture | 6% | Beta pert | Min=4.95%  Max=7.75%  Mode=6% | 4.95% | 7.75% | [44] |
| Incidence of hematoma | 2% | Beta pert | Min=1.28%  Max=2.73%  Mode=2% | 1.28% | 2.73% | [44] |
| Incidence of pneumothorax | 0.5% | Beta pert | Min=0.15%  Max=0.85%  Mode=0.5% | 0.15% | 0.85% | [44] |
| **Utilities** | | | | | | |
| Quality of Life ICU | 0.30 | Beta | s.e.=0.03 | 0.24 | 0.36 | Assumption |
| Quality of Life hospital | 0.60 | Beta | s.e.=0.06 | 0.48 | 0.71 | [56] |
| Quality of Life First 5 years after discharge | 0.67 | Beta | s.e.=0.023 | 0.62 | 0.71 | [45] |
| Quality of Life 5-10 years after discharge | 0.70 | Beta | s.e.=0.025 | 0.65 | 0.75 | [45] |
| Quality of Life >10 years after discharge | 0.68 | Beta | s.e.=0.031 | 0.62 | 0.74 | [45] |
| **Unit costs (2019 Euros)** | | | | | | |
| Analytics (annual) | €959 |  |  | €100 | €20.000 | [47] |
| ICU day | €670.4 | Gamma | s.e.=335.2 | €565.9 | €1,469.5 | [48,57,58] |
| Hospital day | €297.6 | Gamma | s.e.=148.8 | €81.1 | €652.2 | [49] |
| Antibiotics for CRBSI per day | €114.4 | Beta pert | Min=€85.2  Max=€137.4  Mode=€114.4 | €85.2 | €137.4 | [46] |
| Catheter replacement | €17.7 | Gamma | s.e.=€8.9 | 4.8 | 38.9 | [58] |
| Catheter | €12. 6 | Gamma | s.e.=€6.3 | 3.4 | 27.6 | [59] |
| Treatment of arterial puncture | €10.1 | Gamma | s.e.=€5.0 | 2.7 | 22.1 | [60] |
| Treatment of hematoma | €0 | Beta pert | Min= €0  Max=€50  Mode=€0 | 0 | 50 | [61] |
| Treatment of pneumothorax | €96.1 | Gamma | s.e.=€48.0 | 26.2 | 210.6 | [61] |
| **Resource Use** | | | | | | |
| Duration of infection (days) | 10.5 | Beta pert | Min=7  Max=14  Mode=10.5 | 7 | 14 | [46] |
| LOS ICU without CRBSI | 3 | Gamma | s.e.=0.6 | 1.9 | 4.3 | [38] |
| LOS ICU with CRBSI | 13 | Gamma | s.e.=2.6 | 8.4 | 18.6 | [38] |
| Relative change in ICU LOS with intervention | 0.76 | Normal | Se=0.08 | 61% | 100% | [39] |
| LOS hospital after ICU discharge | 15 | Gamma | s.e.=3 | 9.7 | 21.43 | [35,38] |

**References**

1 Condoluci A, Terzi di Bergamo L, Langerbeins P, Hoechstetter MA, Herling CD, De Paoli L, Delgado J, Rabe KG, Gentile M, Doubek M, Mauro FR. International prognostic score for asymptomatic early-stage chronic lymphocytic leukemia. Blood, The Journal of the American Society of Hematology. 2020 May 21;135(21):1859-69.

2 Pflug N, Bahlo J, Shanafelt TD, Eichhorst BF, Bergmann MA, Elter T, Bauer K, Malchau G, Rabe KG, Stilgenbauer S, Döhner H. Development of a comprehensive prognostic index for patients with chronic lymphocytic leukemia. Blood, The Journal of the American Society of Hematology. 2014 Jul 3;124(1):49-62.

3 International CLL-IPI Working Group. An international prognostic index for patients with chronic lymphocytic leukaemia (CLL-IPI): a meta-analysis of individual patient data. The Lancet Oncology. 2016 Jun 1;17(6):779-90.

4 Langerbeins P, Bahlo J, Rhein C, Gerwin H, Cramer P, Fürstenau M, Al‐Sawaf O, von Tresckow J, Fink AM, Kreuzer K, Vehling‐Kaiser U. IBRUTINIB VERSUS PLACEBO IN PATIENTS WITH ASYMPTOMATIC, TREATMENT‐NAÏVE EARLY STAGE CLL: PRIMARY ENDPOINT RESULTS OF THE PHASE 3 DOUBLE‐BLIND RANDOMIZED CLL12 TRIAL. Hematological Oncology. 2019 Jun;37:38-40.

5 TLV The Dental and Pharmaceuticals Benefits Agency. 2020 Retrieved on 26-1-2020 from <https://www.tlv.se/download/18.2b305b2817457f407a2a598b/1599732770817/bes200827_underlag_venclyxto.pdf>

6 Guyot P, Ades AE, Ouwens MJ, Welton NJ. Enhanced secondary analysis of survival data: reconstructing the data from published Kaplan-Meier survival curves. BMC medical research methodology. 2012 Dec;12(1):1-3.

7 WHO mortality database: Query the online database. [http://apps.who.int/healthinfo/statistics/mortality/causeofdeath_query/start.php. Updated 20172016](http://apps.who.int/healthinfo/statistics/mortality/causeofdeath_query/start.php.%20Updated%2020172016).

8 Population pyramids of the world from 1950 to 2100. <https://www.populationpyramid.net/sweden/2016/>. Accessed February 21, 2017.

9 Sylvan SE, Asklid A, Johansson H, Klintman J, Bjellvi J, Tolvgård S, Kimby E, Norin S, Andersson PO, Karlsson C, Karlsson K. First-line therapy in chronic lymphocytic leukemia: a Swedish nation-wide real-world study on 1053 consecutive patients treated between 2007 and 2013. haematologica. 2019 Apr;104(4):797.

10 Svenska KLL gruppen: 2020 Retrieved on 12-1-2020 from <https://kunskapsbanken.cancercentrum.se/diagnoser/kll/>

11 Holtzer-Goor KM, Schaafsma MR, Joosten P, Posthuma EF, Wittebol S, Huijgens PC, Mattijssen EJ, Vreugdenhil G, Visser H, Peters WG, Erjavec Z. Quality of life of patients with chronic lymphocytic leukaemia in the Netherlands: results of a longitudinal multicentre study. Quality of Life Research. 2015 Dec;24(12):2895-906.

12 Kosmas CE, Shingler SL, Samanta K, Wiesner C, Moss PA, Becker U, Lloyd AJ. Health state utilities for chronic lymphocytic leukemia: importance of prolonging progression-free survival. Leukemia & lymphoma. 2015 May 4;56(5):1320-6.

13 Schwarze K, Buchanan J, Fermont JM, Dreau H, Tilley MW, Taylor JM, Antoniou P, Knight SJ, Camps C, Pentony MM, Kvikstad EM. The complete costs of genome sequencing: a microcosting study in cancer and rare diseases from a single center in the United Kingdom. Genetics in Medicine. 2020 Jan;22(1):85-94.

14 Krijkamp EM, Alarid-Escudero F, Enns EA, Pechlivanoglou P, Hunink MM, Yang A, Jalal HJ. A multidimensional array representation of state-transition model dynamics. Medical Decision Making. 2020 Feb;40(2):242-8.

15 Kutsch N, Bahlo J, Robrecht S, Franklin J, Zhang C, Maurer C, De Silva N, Lange E, Weide R, Kiehl MG, Sökler M. Long Term Follow-up Data and Health-Related Quality of Life in Frontline Therapy of Fit Patients Treated With FCR Versus BR (CLL10 Trial of the GCLLSG). HemaSphere. 2020 Feb;4(1).

16 Jeyakumaran D, Kempel A, Côté S. An assessment of the number of chronic lymphocytic leukemia (CLL) patients eligible for front-line treatment but unsuitable for full-dose fludarabine across the European Union. Value in Health. 2016 Nov 1;19(7):A574-5.

17 Goede V, Fischer K, Busch R, Engelke A, Eichhorst B, Wendtner CM, Chagorova T, De La Serna J, Dilhuydy MS, Illmer T, Opat S. Obinutuzumab plus chlorambucil in patients with CLL and coexisting conditions. New England Journal of Medicine. 2014 Mar 20;370(12):1101-10.

18 Fischer K, Al-Sawaf O, Bahlo J, Fink AM, Tandon M, Dixon M, Robrecht S, Warburton S, Humphrey K, Samoylova O, Liberati AM. Venetoclax and obinutuzumab in patients with CLL and coexisting conditions. New England Journal of Medicine. 2019 Jun 6;380(23):2225-36.

19 Emond B, Sundaram M, Romdhani H, Lefebvre P, Wang S, Mato A. Comparison of Time to Next Treatment, Health Care Resource Utilization, and Costs in Patients with Chronic Lymphocytic Leukemia Initiated on Front-line Ibrutinib or Chemoimmunotherapy. Clinical Lymphoma Myeloma and Leukemia. 2019 Dec 1;19(12):763-75.

20 Campo E, Cymbalista F, Ghia P, Jäger U, Pospisilova S, Rosenquist R, Schuh A, Stilgenbauer S. TP53 aberrations in chronic lymphocytic leukemia: an overview of the clinical implications of improved diagnostics. Haematologica. 2018 Dec;103(12):1956.

21 Seymour JF, Kipps TJ, Eichhorst B, Hillmen P, D’Rozario J, Assouline S, Owen C, Gerecitano J, Robak T, De la Serna J, Jaeger U. Venetoclax–rituximab in relapsed or refractory chronic lymphocytic leukemia. New England Journal of Medicine. 2018 Mar 22;378(12):1107-20

22 Eichhorst B, Robak T, Montserrat E, Ghia P, Niemann CU, Kater AP et al. Chronic lymphocytic leukemia: ESMO Clinical Practice Guidelines for diagnosis, treatment and follow-up. Annals of Oncology. 2020. In Press.

23 Munir T, Brown JR, O'Brien S, Barrientos JC, Barr PM, Reddy NM, Coutre S, Tam CS, Mulligan SP, Jaeger U, Kipps TJ. Final analysis from RESONATE: Up to six years of follow‐up on ibrutinib in patients with previously treated chronic lymphocytic leukemia or small lymphocytic lymphoma. American journal of hematology. 2019 Dec;94(12):1353-63.

24 Hallek M, Fischer K, Fingerle-Rowson G, Fink AM, Busch R, Mayer J, Hensel M, Hopfinger G, Hess G, Von Grünhagen U, Bergmann M. Addition of rituximab to fludarabine and cyclophosphamide in patients with chronic lymphocytic leukaemia: a randomised, open-label, phase 3 trial. The Lancet. 2010 Oct 2;376(9747):1164-74.

25 Jones J, Mato A, Coutre S, Byrd JC, Furman RR, Hillmen P, Osterborg A, Tam C, Stilgenbauer S, Wierda WG, Heerema NA. Evaluation of 230 patients with relapsed/refractory deletion 17p chronic lymphocytic leukaemia treated with ibrutinib from 3 clinical trials. British journal of haematology. 2018 Aug;182(4):504-12.

26 Buchanan J, Wordsworth S, Clifford R, Robbe P, Taylor JC, Schuh A, Knight SJ. Using genomic information to guide ibrutinib treatment decisions in chronic lymphocytic leukaemia: a cost-effectiveness analysis. PharmacoEconomics. 2017 Aug;35(8):845-58.

27 Ghatnekar O, Hjalte F, Taylor M. Cost-effectiveness of dasatinib versus high-dose imatinib in patients with Chronic Myeloid Leukemia (CML), resistant to standard dose imatinib–a Swedish model application. Acta Oncologica. 2010 Aug 1;49(6):851-8.

28 Glenngård AH, Persson U, Söderman C. Costs associated with blood transfusions in Sweden–the societal cost of autologous, allogeneic and perioperative RBC transfusion. Transfusion medicine. 2005 Aug;15(4):295-306.

29 Sorensen SV, Peng S, Dorman E, Cote S, Tambour M, Pan F, Sengupta N. The cost-effectiveness of ibrutinib in treatment of relapsed or refractory chronic lymphocytic leukemia. Health Economics & Outcome Research: Open Access. 2016;2(4):1-9.

30 Furman RR, Sharman JP, Coutre SE, Cheson BD, Pagel JM, Hillmen P, Barrientos JC, Zelenetz AD, Kipps TJ, Flinn I, Ghia P. Idelalisib and rituximab in relapsed chronic lymphocytic leukemia. New England Journal of Medicine. 2014 Mar 13;370(11):997-1007.

31 Apostolopoulou E, Raftopoulos V, Filntisis G, et al. Surveillance of device-associated infection rates and mortality in 3 greek intensive care units. Am J Crit Care. 2013;22(3):e12-20.

32 Blot SI, Depuydt P, Annemans L, et al. Clinical and economic outcomes in critically ill patients with nosocomial catheter-related bloodstream infections. Clin Infect Dis. 2005;41(11):1591-1598.

33 Blot S, Poulakou G, Timsit JF. Catheter-associated bloodstream infection rates: how low can you go? 2019

34 Stevens V, Geiger K, Concannon C, Nelson R, Brown J, Dumyati G. Inpatient costs, mortality and 30‐day re‐admission in patients with central‐line‐associated bloodstream infections. Clinical Microbiology and Infection. 2014;20(5).

35 GiViT, Gruppo Italiano per la Valutazione degli Interventi In Terapia Intensiva. Report PROSAFE project; 2014. Report nr Centre GR001.

36 Henry KE, Hager DN, Pronovost PJ, Saria S. A targeted real-time early warning score (TREWScore) for septic shock. Sci Transl Med. 2015;7(299):299ra122.

37 Gardner-Thorpe J, Love N, Wrightson J, Walsh S, Keeling N. The value of modified early warning score (MEWS) in surgical in-patients: A prospective observational study. The Annals of The Royal College of Surgeons of England. 2006;88(6):571-575.

38 van Vught LA, Klouwenberg PMK, Spitoni C, et al. Incidence, risk factors, and attributable mortality of secondary infections in the intensive care unit after admission for sepsis. JAMA. 2016;315(14):1469-1479.

39 Ferrer R, Martin-Loeches I, Phillips G, et al. Empiric antibiotic treatment reduces mortality in severe sepsis and septic shock from the first hour: Results from a guideline-based performance improvement program. Crit Care Med. 2014;42(8):1749-1755.

40 Williams TA, Dobb GJ, Finn JC, Knuiman MW, Geelhoed E, Lee KY, et al. Determinants of long-term survival after intensive care. Crit Care Med 2008; 36:1523-30.

41 Dick A, Liu H, Zwanziger J, et al. Long-term survival and healthcare utilization outcomes attributable to sepsis and pneumonia. BMC health services research. 2012;12(1):1.

42 World Health Organization: WHO Mortality Database: Query the Online Database. Available at: <http://apps.who.int/healthinfo/statistics/mortality/causeofdeath_query/start.php>. Accessed June 12, 2017.

43 Index Mundi: Greece Age structure. Available at: <http://www.indexmundi.com/greece/age_structure.html>. Accessed June 12, 2017

44 Tsotsolis N, Tsirgogianni K, Kioumis I, et al. Pneumothorax as a complication of central venous catheter insertion. Ann Transl Med. 2015;3(3):40-5839.2015.02.11.

45 Cuthbertson BH, Elders A, Hall S, Taylor J, MacLennan G, Mackirdy F, et al. Mortality and quality of life in the five years after severe sepsis. Crit Care 2013; 17:1.

46 Vandijck DM, Depaemelaere M, Labeau SO, et al. Daily cost of antimicrobial therapy in patients with intensive care unit-acquired, laboratory-confirmed bloodstream infection. Int J Antimicrob Agents. 2008;31(2):161-165.

47 Datamed: EKG Format Translators. Product Price List: Effective 4/1/2017. Available at: <http://www.datamed.com/docs/datamed_product_price_list.pdf>. Accessed June 12, 2017

48 Leftakis A, Geitona M. Cost analysis and estimation of thoracic surgical patients with lung cancer in Greece: The case of Sotiria ICU. Intensive Crit Care Nurs 2001; 17:322-30.

49 Geitona M, Hatzikou M, Steiropoulos P, Alexopoulos EC, Bouros D. The cost of COPD exacerbations: A university hospital–based study in Greece. Respir Med 2011; 105:402-9.

50 Girling A, Lilford R, Cole A, Young T. Headroom approach to device development: Current and future directions. International journal of technology assessment in health care. 2015;31(5):331-8.

51 Woods B, Revill P, Sculpher M, Claxton K. Country-level cost-effectiveness thresholds: initial estimates and the need for further research. Value in Health 2016;19(8):929-935..

52 Makras P, Athanasakis K, Boubouchairopoulou N, Rizou S, Anastasilakis A, Kyriopoulos J, et al. Cost-effective osteoporosis treatment thresholds in Greece. Osteoporosis Int 2015;26(7):1949-1957.

53 Geitona M, Kousoulakou H, Kalogeropoulou M, Mitsiki E, Panitti E, Steiropoulos P. Cost-effectiveness analysis of the fixed combination indacaterol/glycopyrronium vs. tiotropium and salmeterol/fluticasone in the management of COPD in Greece. Value in Health 2015;18(7):A500.

54 Jacob V, Thota AB, Chattopadhyay SK, Njie GJ, Proia KK, Hopkins DP, Ross MN, Pronk NP, Clymer JM. Cost and economic benefit of clinical decision support systems for cardiovascular disease 55 prevention: a community guide systematic review. Journal of the American Medical Informatics Association. 2017 May 1;24(3):669-76.

55 Van Calster B, Wynants L, Timmerman D, Steyerberg EW, Collins GS. Predictive analytics in health care: how can we know it works?. Journal of the American Medical Informatics Association. 2019 Dec;26(12):1651-4

56 Cox CE, Carson SS, Govert JA, Chelluri L, Sanders GD. An economic evaluation of prolonged mechanical ventilation. Crit Care Med 2007; 35:1918-27.

57 Karabatsou D, Tsironi M, Tsigou E, Boutzouka E, Katsoulas T, Baltopoulos G. Variable cost of ICU care, a micro-costing analysis. Intensive and Critical Care Nursing. 2016;35:66-73.

58 Hakkaart-van Roijen L, van der Linden N, Bouwmans C, Kanters T, Tan SS. Bijlage 1. Kostenhandleiding: methodologie van kostenonderzoek en referentieprijzen voor economische evaluaties in de gezondheidszorg. Institute for Medical Technology Assessment, Erasmus Universiteit Rotterdam, Rotterdam; 2015: 1-120 (in Dutch).

59 Lorente L, Lecuona M, Jiménez A, et al. Cost/benefit analysis of chlorhexidine-silver sulfadiazine-impregnated venous catheters for femoral access. Am J Infect Control. 2014;42(10):1130-1132.

60 Moretti R, Moretti F. Ultrasound-guided internal jugular access: Systematic review and economic evaluation of cost-effectiveness. OA Evidence-Based Medicine. 2013;1(2):11.

61 Boland A, Haycox A, Bagust A, Fitzsimmons L. A randomised controlled trial to evaluate the clinical and cost-effectiveness of hickman line insertions in adult cancer patients by nurses. NCCHTA; 2003.
